# Analysis of an improved fractional-order model of boundary formation in the *Drosophila* large intestine dependent on Delta-Notch pathway

**DOI:** 10.1186/s13662-020-02836-1

**Published:** 2020-07-23

**Authors:** Deshun Sun, Lingyun Lu, Fei Liu, Li Duan, Daping Wang, Jianyi Xiong

**Affiliations:** 1 Shenzhen Key Laboratory of Tissue Engineering, Shenzhen Laboratory of Digital Orthopedic Engineering, Shenzhen Second People’s Hospital, The First Hospital Affiliated to Shenzhen University, Health Science Center, Shenzhen, 518035 P.R. China; 2 Shenzhen Institute of Advanced Technology, Chinese Academy of Sciences, Shenzhen, 518035 P.R. China; 3 Nanjing Research Institute of Electronic Engineering, Nanjing, 210007 P.R. China; 4 School of Software Engineering, South China University of Technology, Building B7, 510006 Guangzhou, P.R. China

**Keywords:** Delta-Notch signaling pathway, Fractional-order differential equations, Local stability analysis, Sensitive analysis

## Abstract

In this paper, an improved fractional-order model of boundary formation in the *Drosophila* large intestine dependent on Delta-Notch pathway is proposed for the first time. The uniqueness, nonnegativity, and boundedness of solutions are studied. In a two cells model, there are two equilibriums (no-expression of Delta and normal expression of Delta). Local asymptotic stability is proved for both cases. Stability analysis shows that the orders of the fractional-order differential equation model can significantly affect the equilibriums in the two cells model. Numerical simulations are presented to illustrate the conclusions. Next, the sensitivity of model parameters is calculated, and the calculation results show that different parameters have different sensitivities. The most and least sensitive parameters in the two cells model and the 60 cells model are verified by numerical simulations. What is more, we compare the fractional-order model with the integer-order model by simulations, and the results show that the orders can significantly affect the dynamic and the phenotypes.

## Introduction

The *Drosophila* large intestine occupies a major middle portion of the hindgut and is subdivided into dorsal and ventral domains with distinct cell types, and a one-cell-wide strand of boundary cells is induced between them for wild-type embryos. Takashima et al. [1] reported that the identity and localization of boundary cells are mainly determined by Delta, Notch, and activated Notch genes.

For such developmental patterning problems, computational approaches are breaking new ground in understanding how embryos form. Different kinds of computational strategies [2, 3] have been proposed. For example, in 2002, Matsuno et al. [4] analyzed the mechanism of Notch-dependent boundary formation in the *Drosophila* large intestine by genomic object net (GON). Besides, ordinary differential equation (ODE), partial differential equation (PDE), and colored Petri nets are also employed to describe the developmental patterning [5]. The research of the boundary formation in the *Drosophila* large intestine in vivo has been widely explored, but the research in computing is scarce.

Fractional-order systems have been applied in biological systems to better understand the complex behavioral patterns [6–15]. The fractional-order differential equation provided a powerful tool for characterizing memory and hereditary properties of the systems when compared to the integer-order models, and these effects cannot be neglected. For instance, Carla et al. [15] proposed a fractional-order differential equation model to analyze the clinical implications of diabetes mellitus in the dynamics of tuberculosis transmission and proved the stability of disease-free and endemic equilibriums based on the reproduction number. Almeida et al. [11] described the dynamic of SEIR-type epidemics with treatment policies by the fractional-order differential equations. The local asymptotic stability of two equilibriums was proved and the numerical simulations were presented to illustrate the conclusions. In addition, the memory property of the fractional-order differential equation allows the integration of more information from the past, which translates in more accurate predictions for the model. For example, in 2012, Diethelm et al. [8] proposed a fractional-order differential equation model for the simulation of the dynamics of a dengue fever outbreak. By simulations, the author proved that the nonlinear fractional order differential equation model can more accurately simulate the dynamics of infectious diseases than the classical ordinary differential equations. In 2013, Gilberto et al. [9] proposed a nonlinear fractional order model to explore the outbreaks of influenza A(H1N1), and the results showed that the epidemic peak of SEIR fractional epidemic model is more consistent with the peak of the real epidemic data and the mean square error is lower than in the classical model. What is more, in 2020, Lu et al. [16] proposed a fractional-order SEIHDR system to analyze the dynamic behavior of COVID-19. Similarly, the results showed that the fractional-order model also has a better fitting of the data on Beijing, Shanghai, Wuhan, Huanggang, and other cities when compared with the integer-order system. Because of the above-mentioned research, we found that the fractional-order equations may have more potential in application on a real-life system.

With the aforementioned ideas in mind, the Notch signaling pathway is highly conserved in evolution and has significant hereditary properties. Fractional-order differential equation seems much suited for modelling the Notch signal pathway. Therefore, fractional-order differential equations were used to model the mechanism of Notch-dependent boundary formation in the *Drosophila* large intestine.

**Figure 1:**
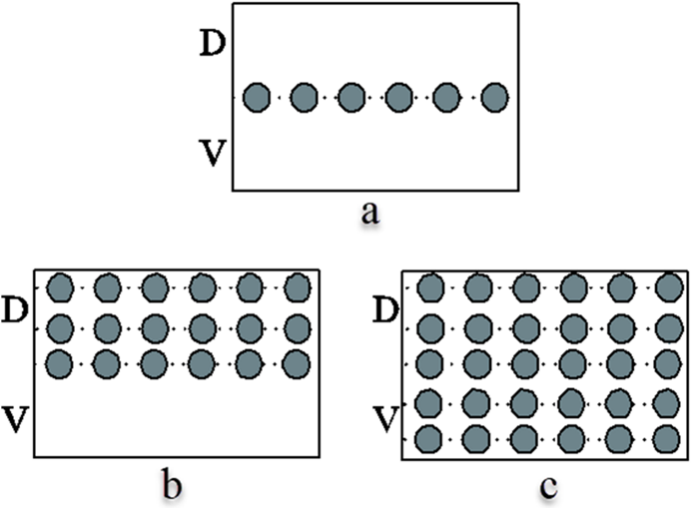
The experimental result of the boundary formation in the *Drosophila* large intestine published in [1, 17, 18]. (**a**) The phenotype of wild type; (**b**) to (**c**) The phenotype of over-expression of Notch. Each filled circle represents a boundary cell. *D* and *V* denote the dorsal and ventral domains, respectively

The purpose of this paper is to analyze the local asymptotic stability of two equilibriums, interpret the experimental results of the boundary cell patterning in the large intestine published in [1, 17, 18] (see Fig. 1), and get the following scenarios (Fig. 1(a)–1(c)) by adjusting sensitive parameters in our model.

## The improved mathematical model

In 2017, our previous work [18] proposed the following model:

1 $$ \textstyle\begin{cases} \frac{dD_{i}}{dt} = \frac{\lambda }{1 + \Delta \cdot A_{i}} - d_{1}D_{i} - \sum_{NG(i)} f_{1} \cdot D_{i},\quad 1 \le i \le NC, \\ \frac{dN_{i}}{dt} = \lambda _{N} - d_{2}N_{i} + \sum_{j \in NG(i)} f_{2} \cdot D_{j} - \frac{aN_{i}}{bD_{i} + N_{i}}, \\ \frac{dA_{i}}{dt} = - d_{3}A_{i} + \frac{aN_{i}}{bD_{i} + N_{i}}, \end{cases} $$

where $D_{i}$, $N_{i}$, and $A_{i}$ represent the concentration of Delta proteins, inactive, and active Notch proteins in *i*th cell, respectively. *λ* is the production of Delta, and Δ is the inhibition coefficient caused by activated Notch. This is because activated Notch can inhibit the production of Delta in the same cell. $d_{i}$, $i = 1,2,3$, means the degradation rate of Delta, inactive and activated Notch. $f_{1}$ denotes the binding rate between the Delta and the neighboring Notch in *i*th cell. Similarly, $f_{2}$ denotes the binding rate between the Notch and the neighboring Delta in *i*th cell. $\lambda _{N}$ denotes the production rate of inactive Notch. *a* represents the transformation rate of Notch proteins from the inactive state to the active state, while *b* describes the inhibition effect of Delta on Notch.

However, Notch signaling pathway is highly conserved in evolution, and the fractional-order differential equation can powerfully characterize memory and hereditary properties of systems when compared to integer-order models. Therefore, the fractional differential equations are employed to model the Notch signal pathway in this paper.

**Figure 2:**
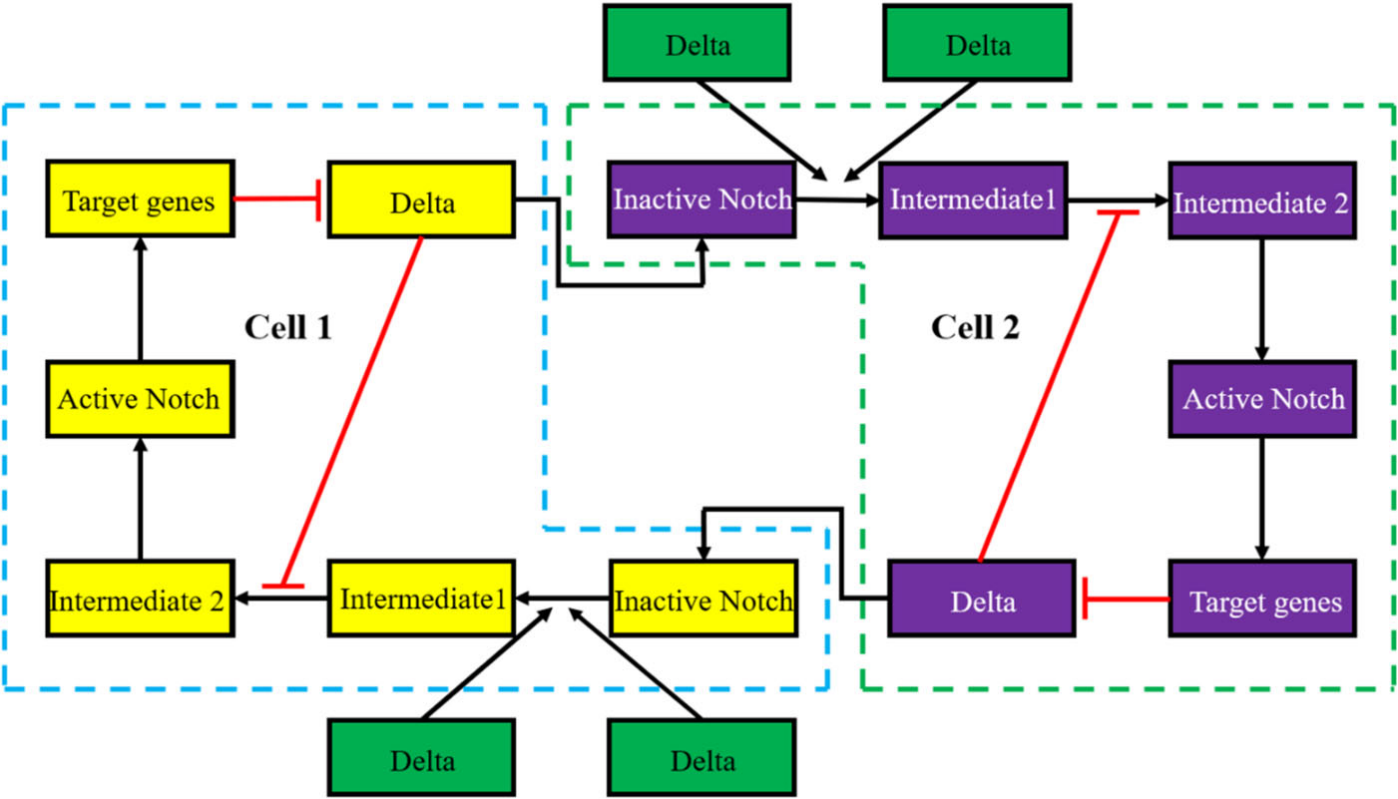
The mechanism of Delta-Notch signaling pathway in two cells

According to the mechanism of Delta-Notch signaling pathway in two cells (Fig. 2), when a Delta ligand binds to the neighboring Notch in *i*th cell, the binding rate is related to the concentration of the Notch receptor; therefore, we use $\sum_{j \in NG(i)} f_{1}D_{i}N_{j}$ instead of the former $\sum_{j \in NG(i)} f_{1}D_{i}$. Similarly, we change $\sum_{j \in NG(i)} f_{1}N_{i}$ into $\sum_{j \in NG(i)} f_{1}D_{j}N_{i}$. What is more, if the production rate of active Notch is $\frac{aN_{i}}{bD_{i} + N_{i}}$, and when the expression of Delta is 0, the concentration of active Notch is $\frac{a}{d_{3}}$ in two cells. This is a contradiction. Because if there is no Delta ligand, the concentration of active Notch will be 0 in biological knowledge. Therefore, compared to $\frac{aN_{i}}{bD_{i} + N_{i}}$, $\frac{a ( \sum_{j \in NG(i)} D_{j}N_{i} )}{b + ( \sum_{j \in NG(i)} D_{j}N_{i} )}$ is more appropriate.

Thus, an improved model based on fractional-order differential equations was proposed as follows:

2 $$ \textstyle\begin{cases} \frac{d^{\alpha } D_{i}}{dt} = \frac{\lambda ^{\alpha }}{1 + \Delta ^{\alpha } A_{i}} - \sum_{j \in NG(i)} f_{1}^{\alpha } D_{i}N_{j} - d^{\alpha } D_{i},\quad 1 \le i \le NC, \\ \frac{d^{\alpha } N_{i}}{dt} = \lambda _{N}^{\alpha } + \sum_{j \in NG(i)} f_{1}^{\alpha } D_{j}N_{i} - d^{\alpha } N_{i}, \\ \frac{d^{\alpha } A_{i}}{dt} = \frac{a^{\alpha } ( \sum_{j \in NG(i)} D_{j}N_{i} )}{b^{\alpha } + ( \sum_{j \in NG(i)} D_{j}N_{i} )} - d^{\alpha } A_{i}, \end{cases} $$

where *α* ($0 < \alpha \le 1$) is the order of the fractional derivative. $\frac{d^{\alpha } D_{i}}{dt}$, $\frac{d^{\alpha } N_{i}}{dt}$, and $\frac{d^{\alpha } A_{i}}{dt}$ denote the Caputo fractional derivative. For example, the Caputo fractional derivative of $\frac{d^{\alpha } D_{i}}{dt}$ is defined as follows:

3 $$ \frac{d^{\alpha } D_{i}}{dt} = I^{n - \alpha } \frac{d^{n}D_{i}}{dt^{n}} = \frac{1}{\varGamma (n - \alpha )} \int _{0}^{t} (t - s)^{(n - \alpha - 1)}D_{i}^{(n)}(s) \,ds, $$

where $n - 1 < \alpha < n$, $n \in \mathbb{N}$ and $\varGamma (\bullet )$ is the gamma function. When $0 < \alpha < 1$,

4 $$ \frac{d^{\alpha } D_{i}}{dt} = \frac{1}{\varGamma (1 - \alpha )} \int _{0}^{t} \frac{D'_{i}(s)}{(t - s)^{\alpha }} \,ds. $$

Biologically speaking, $\frac{d^{\alpha } D_{i}}{dt}$, $\frac{d^{\alpha } N_{i}}{dt}$, and $\frac{d^{\alpha } A_{i}}{dt}$ represent the change rate of the concentration of Delta proteins, inactive and active Notch proteins with hereditary properties.

## Well-posedness

In the following, the well-posedness (uniqueness, nonnegativity, and boundedness of solutions) of two cells is studied.

The model of system (2) has *NC* cells with $3 \times NC$ differential equations. As a result, it is impossible to analyze such a big system in theory. However, we can analyze two cells in theory and map into high dimensional equations. Therefore, the dynamic characteristic of two cells is explored.

Firstly, based on system (2), the model of two cells is proposed according to Fig. 2:

5 $$ \textstyle\begin{cases} \frac{d^{\alpha } D_{1}}{dt} = \frac{\lambda ^{\alpha }}{1 + \theta ^{\alpha } A_{1}} - f^{\alpha } D_{1}N_{2} - d^{\alpha } D_{1}, \\ \frac{d^{\alpha } N_{1}}{dt} = \lambda _{N}^{\alpha } + f^{\alpha } D_{2}N_{1} - d^{\alpha } N_{1}, \\ \frac{d^{\alpha } A_{1}}{dt} = \frac{a^{\alpha } D_{2}N_{1}}{b^{\alpha } + D_{2}N_{1}} - d^{\alpha } A_{1}, \\ \frac{d^{\alpha } D_{2}}{dt} = \frac{\lambda ^{\alpha }}{1 + \theta ^{\alpha } A_{2}} - f^{\alpha } D_{2}N_{1} - d^{\alpha } D_{2}, \\ \frac{d^{\alpha } N_{2}}{dt} = \lambda _{N}^{\alpha } + f^{\alpha } D_{1}N_{2} - d^{\alpha } N_{2}, \\ \frac{d^{\alpha } A_{2}}{dt} = \frac{a^{\alpha } D_{1}N_{2}}{b^{\alpha } + D_{1}N_{2}} - d^{\alpha } A_{2}. \end{cases} $$

### Nonnegativity and boundedness

Firstly, we prove that $D_{1}(t) \ge 0$, $\forall t \ge 0$, assuming $D_{1}(0) > 0$ for $t = 0$. Let us suppose that $D_{1}(t) \ge 0$, $\forall t \ge 0$ is not true. Thus, there exists $t_{1} > 0$ such that $D_{1}(t) > 0$ for $0 \le t < t_{1}$, $D_{1}(t_{1}) = 0$, and $D_{1}(t) < 0$ for $t > t_{1}$.

From the first equation of (5), we have $\frac{d^{\alpha } D_{1}(t)}{dt}| _{ t = t_{1}} > 0$. Based on Corollary 1 of [19], we get $D_{1}(t_{1}^{ +} ) > 0$, which contradicts the fact $D_{1}(t_{1}^{ +} ) < 0$. Therefore, we have $D_{1}(t) \ge 0$, $\forall t \ge 0$. Using the same arguments, $N_{1}(t) \ge 0$, $A_{1}(t) \ge 0$, $D_{2}(t) \ge 0$, $N_{2}(t) \ge 0$, $A_{2}(t) \ge 0$, $\forall t \ge 0$. Next, we will prove the boundedness.

We define a function $w(t) = D_{1}(t) + N_{1}(t) + A_{1}(t) + D_{2}(t) + N_{2}(t) + A_{2}(t)$. From equation (5), we obtain

$$\begin{aligned}& \frac{d^{\alpha } w(t)}{dt} + \delta w(t) \\& \quad = \frac{\lambda ^{\alpha }}{1 + \theta ^{\alpha } A_{1}} - f^{\alpha } D_{1}N_{2} - d^{\alpha } D_{1} + \lambda _{N}^{\alpha } + f^{\alpha } D_{2}N_{1} - d^{\alpha } N_{1} + \frac{a^{\alpha } D_{2}N_{1}}{b^{\alpha } + D_{2}N_{1}} \\& \quad\quad{} - d^{\alpha } A_{1} + \frac{\lambda ^{\alpha }}{1 + \theta ^{\alpha } A_{2}} - f^{\alpha } D_{2}N_{1} - d^{\alpha } D_{2} + \lambda _{N}^{\alpha } + f^{\alpha } D_{1}N_{2} - d^{\alpha } N_{2} + \frac{a^{\alpha } D_{1}N_{2}}{b^{\alpha } + D_{1}N_{2}} \\& \quad\quad{} - d^{\alpha } A_{2} + \delta D_{1}(t) + \delta N_{1}(t) + \delta A_{1}(t) + \delta D_{2}(t) + \delta N_{2}(t) + \delta A_{2}(t) \\& \quad \le 2\lambda ^{\alpha } + 2\lambda _{N}^{\alpha } + 2a^{\alpha } + \bigl(\delta - d^{\alpha } \bigr) \bigl(D_{1}(t) + N_{1}(t) + A_{1}(t) + D_{2}(t) + N_{2}(t) + A_{2}(t)\bigr). \end{aligned}$$

Taking $\delta = d^{\alpha } $, $\frac{d^{\alpha } w(t)}{dt} + \delta w(t) \le 2\lambda ^{\alpha } + 2\lambda _{N}^{\alpha } + 2a^{\alpha } $. Based on [20], the boundedness is proved.

### Existence and uniqueness

Consider a mapping $F(X) = (F_{1}(X),F_{2}(X),F_{3}(X),F_{4}(X),F_{5}(X),F_{6}(X))$, where

$$\begin{aligned}& X = \begin{bmatrix} D_{1} \\ N_{1} \\ A_{1} \\ D_{2} \\ N_{2} \\ A_{2} \end{bmatrix}, \quad\quad \bar{X} = \begin{bmatrix} \bar{D}_{1} \\ \bar{N}_{1} \\ \bar{A}_{1} \\ \bar{D}_{2} \\ \bar{N}_{2} \\ \bar{A}_{2} \end{bmatrix},\quad \text{and} \quad \textstyle\begin{cases} F_{1}(X) = \frac{\lambda ^{\alpha }}{1 + \theta ^{\alpha } A_{1}} - f^{\alpha } D_{1}N_{2} - d^{\alpha } D_{1}, \\ F_{2}(X) = \lambda _{N}^{\alpha } + f^{\alpha } D_{2}N_{1} - d^{\alpha } N_{1}, \\ F_{3}(X) = \frac{a^{\alpha } D_{2}N_{1}}{b^{\alpha } + D_{2}N_{1}} - d^{\alpha } A_{1}, \\ F_{4}(X) = \frac{\lambda ^{\alpha }}{1 + \theta ^{\alpha } A_{2}} - f^{\alpha } D_{2}N_{1} - d^{\alpha } D_{2}, \\ F_{5}(X) = \lambda _{N}^{\alpha } + f^{\alpha } D_{1}N_{2} - d^{\alpha } N_{2}, \\ F_{6}(X) = \frac{a^{\alpha } D_{1}N_{2}}{b^{\alpha } + D_{1}N_{2}} - d^{\alpha } A_{2}, \end{cases}\displaystyle \end{aligned}$$

then we have

$$\begin{aligned} & \bigl\Vert F(X) - F(\bar{X}) \bigr\Vert \\ & \quad = \biggl\vert \frac{\lambda ^{\alpha }}{1 + \theta ^{\alpha } A_{1}} - \frac{\lambda ^{\alpha }}{1 + \theta ^{\alpha } \bar{A}_{1}} - f^{\alpha } (D_{1}N_{2} - \bar{D}_{1} \bar{N}_{2}) - d^{\alpha } (D_{1} - \bar{D}_{1}) \biggr\vert \\ & \quad \quad {} + \bigl\vert f^{\alpha } (D_{2}N_{1} - \bar{D}_{2}\bar{N}_{1}) - d^{\alpha } (N_{1} - \bar{N}_{1}) \bigr\vert \\ & \quad\quad {} + \biggl\vert \frac{a^{\alpha } D_{2}N_{1}}{b^{\alpha } + D_{2}N_{1}} - \frac{a^{\alpha } \bar{D}_{2}\bar{N}_{1}}{b^{\alpha } + \bar{D}_{2}\bar{N}_{1}} - d^{\alpha } (A_{1} - \bar{A}_{1}) \biggr\vert \\ & \quad\quad {} + \biggl\vert \frac{\lambda ^{\alpha }}{1 + \theta ^{\alpha } A_{2}} - \frac{\lambda ^{\alpha }}{1 + \theta ^{\alpha } \bar{A}_{2}} - f^{\alpha } (D_{2}N_{1} - \bar{D}_{2} \bar{N}_{1}) - d^{\alpha } (D_{2} - \bar{D}_{2}) \biggr\vert \\ & \quad \quad {} + \bigl\vert f^{\alpha } (D_{1}N_{2} - \bar{D}_{1}\bar{N}_{2}) - d^{\alpha } (N_{2} - \bar{N}_{2}) \bigr\vert \\ & \quad \quad {} + \biggl\vert \frac{a^{\alpha } D_{1}N_{2}}{b^{\alpha } + D_{1}N_{2}} - \frac{a^{\alpha } \bar{D}_{1}\bar{N}_{2}}{b^{\alpha } + \bar{D}_{1}\bar{N}_{2}} - d^{\alpha } (A_{2} - \bar{A}_{2}) \biggr\vert \\ & \quad \le \biggl\vert \frac{\lambda ^{\alpha }}{1 + \theta ^{\alpha } A_{1}} - \frac{\lambda ^{\alpha }}{1 + \theta ^{\alpha } \bar{A}_{1}} \biggr\vert + f^{\alpha } \vert D_{1}N_{2} - \bar{D}_{1}\bar{N}_{2} \vert + d^{\alpha } \vert D_{1} - \bar{D}_{1} \vert \\ & \quad \quad {} + f^{\alpha } \vert D_{2}N_{1} - \bar{D}_{2}\bar{N}_{1} \vert + d^{\alpha } \vert N_{1} - \bar{N}_{1} \vert + \biggl\vert \frac{a^{\alpha } D_{2}N_{1}}{b^{\alpha } + D_{2}N_{1}} - \frac{a^{\alpha } \bar{D}_{2}\bar{N}_{1}}{b^{\alpha } + \bar{D}_{2}\bar{N}_{1}} \biggr\vert \\ & \quad \quad {} + d^{\alpha } \vert A_{1} - \bar{A}_{1} \vert + \biggl\vert \frac{\lambda ^{\alpha }}{1 + \theta ^{\alpha } A_{2}} - \frac{\lambda ^{\alpha }}{1 + \theta ^{\alpha } \bar{A}_{2}} \biggr\vert + f^{\alpha } \vert D_{2}N_{1} - \bar{D}_{2}\bar{N}_{1} \vert + d^{\alpha } \vert D_{2} - \bar{D}_{2} \vert \\ & \quad \quad {} + f^{\alpha } \vert D_{1}N_{2} - \bar{D}_{1}\bar{N}_{2} \vert + d^{\alpha } \vert N_{2} - \bar{N}_{2} \vert + \biggl\vert \frac{a^{\alpha } D_{1}N_{2}}{b^{\alpha } + D_{1}N_{2}} - \frac{a^{\alpha } \bar{D}_{1}\bar{N}_{2}}{b^{\alpha } + \bar{D}_{1}\bar{N}_{2}} \biggr\vert + d^{\alpha } \vert A_{2} - \bar{A}_{2} \vert \\ & \quad \le \frac{\lambda ^{\alpha } \theta ^{\alpha }}{(1 + \theta ^{\alpha } A_{1})(1 + \theta ^{\alpha } \bar{A}_{1})} \vert A_{1} - \bar{A}_{1} \vert + Mf^{\alpha } \vert D_{1} - \bar{D}_{1} \vert + Mf^{\alpha } \vert N_{1} - \bar{N}_{1} \vert + d^{\alpha } \vert D_{1} - \bar{D}_{1} \vert \\ & \quad\quad {} + Mf^{\alpha } \vert N_{1} - \bar{N}_{1} \vert + Mf^{\alpha } \vert D_{2} - \bar{D}_{2} \vert + d^{\alpha } \vert N_{1} - \bar{N}_{1} \vert + a^{\alpha } b^{\alpha } \biggl\vert \frac{D_{2}N_{1} - \bar{D}_{2}\bar{N}_{1}}{(b^{\alpha } + D_{2}N_{1})(b^{\alpha } + \bar{D}_{2}\bar{N}_{1})} \biggr\vert \\ & \quad\quad {} + d^{\alpha } \vert A_{1} - \bar{A}_{1} \vert + \frac{\lambda ^{\alpha } \theta ^{\alpha }}{(1 + \theta ^{\alpha } A_{2})(1 + \theta ^{\alpha } \bar{A}_{2})} \vert A_{2} - \bar{A}_{2} \vert + Mf^{\alpha } \vert D_{2} - \bar{D}_{2} \vert + Mf^{\alpha } \vert N_{2} - \bar{N}_{2} \vert \\ & \quad \quad {} + d^{\alpha } \vert D_{2} - \bar{D}_{2} \vert + Mf^{\alpha } \vert N_{2} - \bar{N}_{2} \vert + Mf^{\alpha } \vert D_{1} - \bar{D}_{1} \vert + d^{\alpha } \vert N_{2} - \bar{N}_{2} \vert \\ & \quad \quad {} + a^{\alpha } b^{\alpha } \biggl\vert \frac{D_{1}N_{2} - \bar{D}_{1}\bar{N}_{2}}{(b^{\alpha } + D_{1}N_{2})(b^{\alpha } + \bar{D}_{1}\bar{N}_{2})} \biggr\vert + d^{\alpha } \vert A_{2} - \bar{A}_{2} \vert \\ & \quad \le Mf^{\alpha } \vert D_{1} - \bar{D}_{1} \vert + Mf^{\alpha } \vert D_{1} - \bar{D}_{1} \vert + a^{\alpha } b^{\alpha } M \vert D_{1} - \bar{D}_{1} \vert + d^{\alpha } \vert D_{1} - \bar{D}_{1} \vert \\ & \quad \quad {} + Mf^{\alpha } \vert N_{1} - \bar{N}_{1} \vert + Mf^{\alpha } \vert N_{1} - \bar{N}_{1} \vert + d^{\alpha } \vert N_{1} - \bar{N}_{1} \vert + a^{\alpha } b^{\alpha } M \vert N_{1} - \bar{N}_{1} \vert \\ & \quad \quad {} + \lambda ^{\alpha } \theta ^{\alpha } \vert A_{1} - \bar{A}_{1} \vert + d^{\alpha } \vert A_{1} - \bar{A}_{1} \vert \\ & \quad\quad {} + Mf^{\alpha } \vert D_{2} - \bar{D}_{2} \vert + Mf^{\alpha } \vert D_{2} - \bar{D}_{2} \vert + a^{\alpha } b^{\alpha } M \vert D_{2} - \bar{D}_{2} \vert + d^{\alpha } \vert D_{2} - \bar{D}_{2} \vert \\ & \quad \quad {} + Mf^{\alpha } \vert N_{2} - \bar{N}_{2} \vert + Mf^{\alpha } \vert N_{2} - \bar{N}_{2} \vert + d^{\alpha } \vert N_{2} - \bar{N}_{2} \vert + a^{\alpha } b^{\alpha } M \vert N_{2} - \bar{N}_{2} \vert \\ & \quad \quad {} + \lambda ^{\alpha } \theta ^{\alpha } \vert A_{2} - \bar{A}_{2} \vert + d^{\alpha } \vert A_{2} - \bar{A}_{2} \vert \\ & \quad = \bigl(2Mf^{\alpha } + a^{\alpha } b^{\alpha } M + d^{\alpha } \bigr) \vert D_{1} - \bar{D}_{1} \vert + \bigl(2Mf^{\alpha } + a^{\alpha } b^{\alpha } M + d^{\alpha } \bigr) \vert N_{1} - \bar{N}_{1} \vert \\ & \quad \quad {}+ \bigl(\lambda ^{\alpha } \theta ^{\alpha } + d^{\alpha } \bigr) \vert A_{1} - \bar{A}_{1} \vert \\ & \quad \quad {} + \bigl(2Mf^{\alpha } + a^{\alpha } b^{\alpha } M + d^{\alpha } \bigr) \vert D_{2} - \bar{D}_{2} \vert + \bigl(2Mf^{\alpha } + a^{\alpha } b^{\alpha } M + d^{\alpha } \bigr) \vert N_{2} - \bar{N}_{2} \vert \\ & \quad \quad {}+ \bigl(\lambda ^{\alpha } \theta ^{\alpha } + d^{\alpha } \bigr) \vert A_{2} - \bar{A}_{2} \vert \\ & \quad \le L \Vert X - \bar{X} \Vert , \end{aligned}$$

where $L = \max \{ 2Mf^{\alpha } + a^{\alpha } b^{\alpha } M + d^{\alpha },\lambda ^{\alpha } \theta ^{\alpha } + d^{\alpha } \} $.

Therefore, the existence and uniqueness are proved.

## Equilibriums and stability analysis

In what follows, the equilibriums, stability analysis, and simulations for the two cell model are studied.

### Equilibriums

In this part, the dynamic characteristic of two cells is explored and two scenarios (one is the expression level of Delta is 0, another is not) are considered.

When the expression level of Delta is 0, namely $\lambda = 0$, the equilibrium is

$$ D_{1}^{0} = D_{2}^{0} = 0,\qquad N_{1}^{0} = N_{2}^{0} = \frac{\lambda _{N}^{\alpha }}{d^{\alpha }},\qquad A_{1}^{0} = A_{2}^{0} = 0,\qquad E_{0} = \biggl(0,\frac{\lambda _{N}^{\alpha }}{d^{\alpha }},0,0, \frac{\lambda _{N}^{\alpha }}{d^{\alpha }},0\biggr). $$

When the expression of Delta is normal or over-expression, the equilibrium is

$$\begin{aligned}& E_{1} = \bigl(D_{1}^{1},N_{1}^{1},A_{1}^{1},D_{2}^{1},N_{2}^{1},A_{2}^{1} \bigr),\quad \text{and} \quad D_{1}^{1} = D_{2}^{1} = \frac{d^{\alpha } N_{1}^{1} - \lambda _{N}^{\alpha }}{d^{\alpha } N_{1}^{1}}, \\& A_{1}^{1} = A_{2}^{1} = \frac{a^{\alpha } (d^{\alpha } N_{1}^{1} - \lambda _{N}^{\alpha } )}{d^{\alpha } [ b^{\alpha } d^{\alpha } + (d^{\alpha } N_{1}^{1} - \lambda _{N}^{\alpha } ) ]}, \end{aligned}$$

where $N_{1}^{1} = N_{2}^{1}$ is the solution of equation (6):

6 $$\begin{aligned}& \begin{aligned}[b] & d^{\alpha } f^{\alpha } N^{2} + \bigl(d^{2\alpha } + \lambda _{N}^{\alpha } f^{\alpha } \bigr)N - d^{\alpha } \lambda _{N}^{\alpha } \\ &\quad = \frac{\lambda ^{\alpha } d^{2\alpha } f^{\alpha } N^{2} + \lambda ^{\alpha } d^{\alpha } f^{\alpha } (b^{\alpha } f^{\alpha } - \lambda _{N}^{\alpha } )N}{(d^{2\alpha } + a^{\alpha } \theta ^{\alpha } d^{\alpha } )N + (d^{\alpha } b^{\alpha } f^{\alpha } - d^{\alpha } \lambda _{N}^{\alpha } - a^{\alpha } \theta ^{\alpha } \lambda _{N}^{\alpha } )}. \end{aligned} \end{aligned}$$

Simplify equation (6) and get the following form:

7 $$\begin{aligned} &d^{\alpha } f^{\alpha } \bigl(d^{2\alpha } + a^{\alpha } \theta ^{\alpha } d^{\alpha } \bigr)N^{3} + \bigl[ \bigl(d^{2\alpha } + a^{\alpha } \theta ^{\alpha } d^{\alpha } \bigr) \bigl(d^{2\alpha } + \lambda _{N}^{\alpha } f^{\alpha } \bigr) \\ &\quad{} + d^{\alpha } f^{\alpha } \bigl(d^{\alpha } b^{\alpha } f^{\alpha } - d^{\alpha } \lambda _{N}^{\alpha } - a^{\alpha } \theta ^{\alpha } \lambda _{N}^{\alpha } \bigr) - \lambda ^{\alpha } d^{2\alpha } f^{\alpha } \bigr]N^{2} \\ &\quad{} + \bigl[\bigl(d^{2\alpha } + \lambda _{N}^{\alpha } f^{\alpha } \bigr) \bigl(d^{\alpha } b^{\alpha } f^{\alpha } - d^{\alpha } \lambda _{N}^{\alpha } - a^{\alpha } \theta ^{\alpha } \lambda _{N}^{\alpha } \bigr) - d^{\alpha } \lambda _{N}^{\alpha } \bigl(d^{2\alpha } + a^{\alpha } \theta ^{\alpha } d^{\alpha } \bigr) \\ &\quad{} - \lambda ^{\alpha } d^{\alpha } f^{\alpha } \bigl(b^{\alpha } f^{\alpha } - \lambda _{N}^{\alpha } \bigr)\bigr]N - d^{\alpha } \lambda _{N}^{\alpha } \bigl(d^{\alpha } b^{\alpha } f^{\alpha } - d^{\alpha } \lambda _{N}^{\alpha } - a^{\alpha } \theta ^{\alpha } \lambda _{N}^{\alpha } \bigr) = 0. \end{aligned}$$

Define

8 $$ \begin{gathered} B_{1} = d^{\alpha } f^{\alpha } \bigl(d^{2\alpha } + a^{\alpha } \theta ^{\alpha } d^{\alpha } \bigr), \\ \begin{aligned} B_{2} &= \bigl(d^{2\alpha } + a^{\alpha } \theta ^{\alpha } d^{\alpha } \bigr) \bigl(d^{2\alpha } + \lambda _{N}^{\alpha } f^{\alpha } \bigr) + d^{\alpha } f^{\alpha } \bigl(d^{\alpha } b^{\alpha } f^{\alpha } - d^{\alpha } \lambda _{N}^{\alpha } - a^{\alpha } \theta ^{\alpha } \lambda _{N}^{\alpha } \bigr) \\ &\quad {}- \lambda ^{\alpha } d^{2\alpha } f^{\alpha }, \end{aligned} \\ \begin{aligned} B_{3} &= \bigl(d^{2\alpha } + \lambda _{N}^{\alpha } f^{\alpha } \bigr) \bigl(d^{\alpha } b^{\alpha } f^{\alpha } - d^{\alpha } \lambda _{N}^{\alpha } - a^{\alpha } \theta ^{\alpha } \lambda _{N}^{\alpha } \bigr) - d^{\alpha } \lambda _{N}^{\alpha } \bigl(d^{2\alpha } + a^{\alpha } \theta ^{\alpha } d^{\alpha } \bigr) \\ &\quad{} - \lambda ^{\alpha } d^{\alpha } f^{\alpha } \bigl(b^{\alpha } f^{\alpha } - \lambda _{N}^{\alpha } \bigr), \end{aligned} \\ B_{4} = - d^{\alpha } \lambda _{N}^{\alpha } \bigl(d^{\alpha } b^{\alpha } f^{\alpha } - d^{\alpha } \lambda _{N}^{\alpha } - a^{\alpha } \theta ^{\alpha } \lambda _{N}^{\alpha } \bigr). \end{gathered} $$

Then the equation becomes

9 $$ B_{1}N_{1}^{3} + B_{2}N_{1}^{2} + B_{3}N_{1} + B_{4} = 0. $$

Calculate equation (9) and get the following solution:

10 $$ N_{1} = \sqrt[3]{ - \frac{q}{2} + \sqrt{ \biggl( \frac{q}{2} \biggr)^{2} + \biggl( \frac{p}{3} \biggr)^{3}}} + \sqrt[3]{ - \frac{q}{2} - \sqrt{ \biggl( \frac{q}{2} \biggr)^{2} + \biggl( \frac{p}{3} \biggr)^{3}}} - \frac{B_{2}}{3B_{1}}, $$

where $p = \frac{3B_{1}B_{3} - B_{2}^{2}}{3B_{1}^{2}}$, $q = \frac{27B_{1}^{2}B_{4} - 9B_{1}B_{2}B_{3} + 2B_{2}^{3}}{27B_{1}^{3}}$.

### Stability analysis

In this subsection, the stability of $E_{0}$ and $E_{1}$ is explored [21–24]. Firstly, we compute the Jacobi matrix as follows:

11 $$ \mathit{Jac} = \begin{bmatrix} - f^{\alpha } N - d^{\alpha } & - f^{\alpha } D & - \frac{\theta ^{\alpha } \lambda ^{\alpha }}{(1 + \theta ^{\alpha } A)^{2}} \\ f^{\alpha } N & f^{\alpha } D - d^{\alpha } & 0 \\ \frac{a^{\alpha } b^{\alpha } N}{(b^{\alpha } + DN)^{2}} & \frac{a^{\alpha } b^{\alpha } D}{(b^{\alpha } + DN)^{2}} & - d^{\alpha } \end{bmatrix}. $$

Then, we get the characteristic determinant

12 $$ \bigl\vert S^{\alpha } I - \mathit{Jac} \bigr\vert = \begin{vmatrix} S^{\alpha } + f^{\alpha } N + d^{\alpha } & f^{\alpha } D & \frac{\theta ^{\alpha } \lambda ^{\alpha }}{(1 + \theta A)^{2}} \\ - f^{\alpha } N & S^{\alpha } - f^{\alpha } D + d^{\alpha } & 0 \\ - \frac{a^{\alpha } b^{\alpha } N}{(b^{\alpha } + DN)^{2}} & - \frac{a^{\alpha } b^{\alpha } D}{(b^{\alpha } + DN)^{2}} & S^{\alpha } + d^{\alpha } \end{vmatrix}. $$

Let $\xi = S^{\alpha } $ and when there is no expression of Delta ($\lambda = 0$), the characteristic determinant becomes

13 $$ \bigl(\xi + d^{\alpha } \bigr) \bigl(\xi + d^{\alpha } \bigr) \biggl[ \xi + \frac{\lambda _{N}^{\alpha } f^{\alpha }}{d^{\alpha }} + d^{\alpha } \biggr] = 0 $$

and the corresponding eigenvalues are $\xi _{1,2} = - d^{\alpha }$, $\xi _{3} = - \frac{\lambda _{N}^{\alpha } f^{\alpha }}{d^{\alpha }} - d^{\alpha } $. Obviously, $\vert \arg (S_{1,2,3}) \vert > \frac{\alpha \pi }{2}$. Therefore, when $\lambda = 0$, the equilibrium $E_{0} = (0,\frac{\lambda _{N}^{\alpha }}{d^{\alpha }},0,0,\frac{\lambda _{N}^{\alpha }}{d^{\alpha }},0)$ is locally asymptotically stable [21].

In order to verify the validity of the theoretical analysis results, the numerical simulations have been done. According to our previous work [18], the parameters are shown in Table 1, and the time span is [0, 4000]. The initial values are 0.5, 0.5, 0.5, 0.5, 0.5, 0.5, respectively.

**Table 1:** The parameters for simulation

Parameter	*λ*	*f*	*d*	$\lambda _{N}$	*a*	*b*	*θ*	*α*
Value	0/1000	0.01	0.01	0.07	0.01	200	1e6	0.9

**Figure 3:**
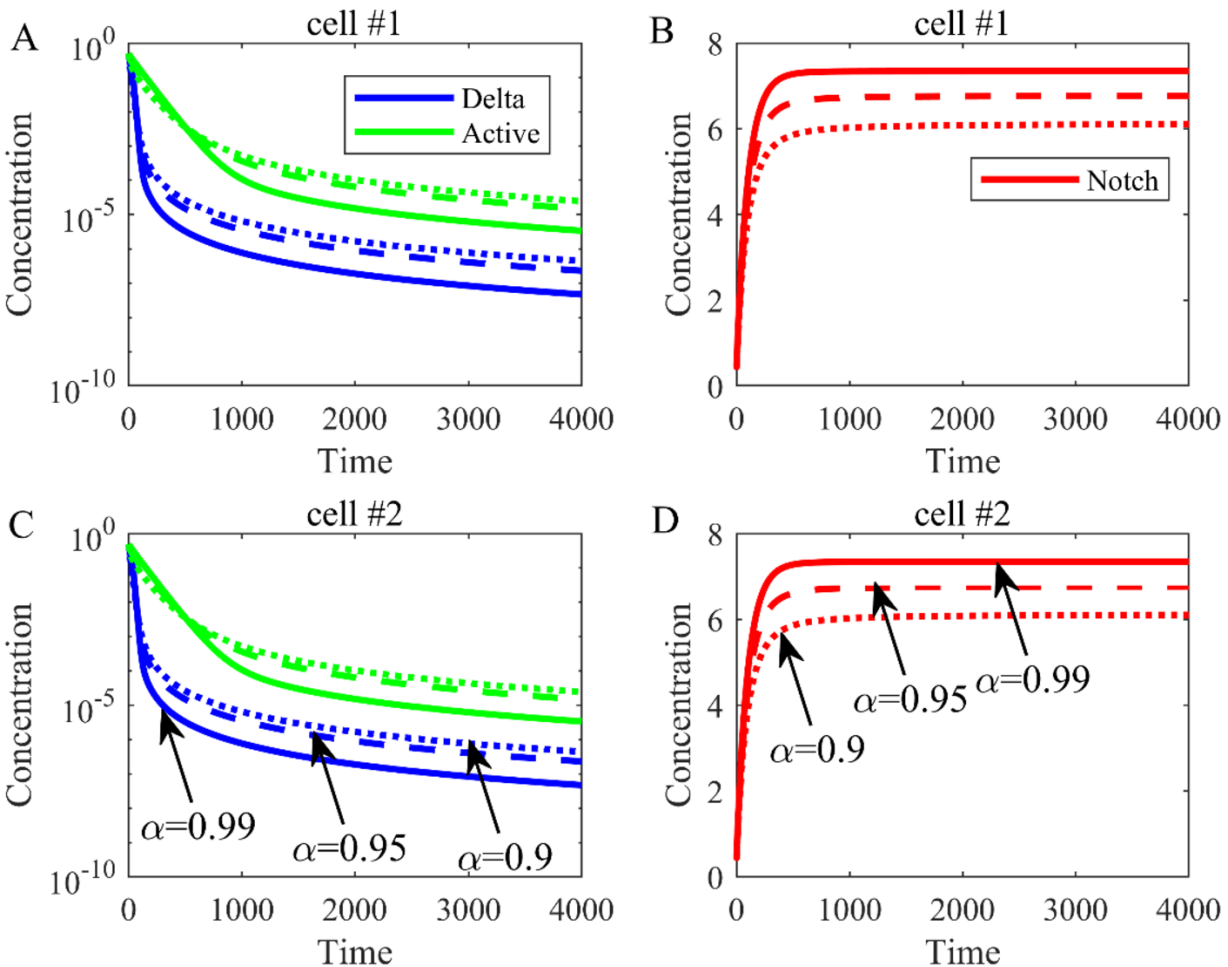
The dynamical trend of Delta, Notch, and active Notch when there is no expression of Delta ($\lambda = 0$). (**A**) The dynamical trend of Delta and active Notch in cell 1. (**B**) The dynamical trend of Notch in cell 1. (**C**) The dynamical trend of Delta and active Notch in cell 2. (**D**) The dynamical trend of Notch in cell 2

Based on the parameters in Table 1, we have calculated the equilibrium $E_{0} = (0,6.0165, 0,0,6.0165,0)$ and the dynamical trends of Delta, Notch, and active Notch are shown when $\lambda = 0$ (Fig. 3). The blue line represents the concentration of Delta, the red line represents the concentration of Notch, and the green line represents the concentration of active Notch. Besides, by changing the order (*α*) of fractional differential equations from 0.9 to 0.99, the trends are the same, but the equilibrium is bigger with the increase of the order.

The numerical solution of system (5) has the following form:

$$ \textstyle\begin{cases}D_{1}(t_{k}) = [ \frac{\lambda ^{\alpha }}{1 + \theta ^{\alpha } A_{1}(t_{k - 1})} - f^{\alpha } D_{1}(t_{k - 1})N_{2}(t_{k - 1}) - d^{\alpha } D_{1}(t_{k - 1}) ]h^{q_{1}} - \sum_{j = v}^{k} c_{j}^{(q_{1})}D_{1}(t_{k - j}), \\ N_{1}(t_{k}) = [ \lambda _{N}^{\alpha } + f^{\alpha } D_{2}(t_{k - 1})N_{1}(t_{k - 1}) - d^{\alpha } N_{1}(t_{k - 1}) ]h^{q_{1}} - \sum_{j = v}^{k} c_{j}^{(q_{1})}N_{1}(t_{k - j}), \\ A_{1}(t_{k}) = [ \frac{a^{\alpha } D_{2}(t_{k - 1})N_{1}(t_{k - 1})}{b^{\alpha } + D_{2}(t_{k - 1})N_{1}(t_{k - 1})} - d^{\alpha } A_{1}(t_{k - 1}) ]h^{q_{1}} - \sum_{j = v}^{k} c_{j}^{(q_{1})}A_{1}(t_{k - j}), \\ D_{2}(t_{k}) = [ \frac{\lambda ^{\alpha }}{1 + \theta ^{\alpha } A_{2}(t_{k - 1})} - f^{\alpha } D_{2}(t_{k - 1})N_{1}(t_{k - 1}) - d^{\alpha } D_{2}(t_{k - 1}) ]h^{q_{1}} - \sum_{j = v}^{k} c_{j}^{(q_{1})}D_{2}(t_{k - j}), \\ N_{2}(t_{k}) = [ \lambda _{N}^{\alpha } + f^{\alpha } D_{1}(t_{k - 1})N_{2}(t_{k - 1}) - d^{\alpha } N_{2}(t_{k - 1}) ]h^{q_{1}} - \sum_{j = v}^{k} c_{j}^{(q_{1})}N_{2}(t_{k - j}), \\ A_{2}(t_{k}) = [ \frac{a^{\alpha } D_{1}(t_{k - 1})N_{2}(t_{k - 1})}{b^{\alpha } + D_{1}(t_{k - 1})N_{2}(t_{k - 1})} - d^{\alpha } A_{2}(t_{k - 1}) ]h^{q_{1}} - \sum_{j = v}^{k} c_{j}^{(q_{1})}A_{2}(t_{k - j}), \end{cases} $$

where $T_{\mathrm{sim}}$ is the simulation time, $k = 1,2,3,\ldots,N$, for $N = [T_{\mathrm{sim}}/h]$, and $(D_{1}(0),N_{1}(0), A_{1}(0),D_{2}(0),N_{2}(0),A_{2}(0))$ is the initial condition. The binomial coefficients $c_{j}^{(q_{i})}$ for ∀*i* are calculated according to the relation $c_{0}^{(q)} = 1$, $c_{j}^{(q)} = ( 1 - \frac{1 + q}{j} )c_{j - 1}^{(q)}$.

Next, the local asymptotic stability at $E_{1} = (D_{1}^{1},N_{1}^{1},A_{1}^{1},D_{2}^{1},N_{2}^{1},A_{2}^{1})$ will be explored.

When $\lambda \ne 0$, the characteristic equation is

14 $$ \begin{aligned}[b] & \xi ^{3} + \bigl(f^{\alpha } N - f^{\alpha } D + 3d^{\alpha } \bigr)\xi ^{2} + \biggl[2d^{\alpha } f^{\alpha } N - 2d^{\alpha } f^{\alpha } D + 3d^{2\alpha } \\ &\quad{} + \frac{a^{\alpha } b^{\alpha } \theta ^{\alpha } \lambda ^{\alpha } N}{(1 + \theta ^{\alpha } A)^{2}(b^{\alpha } + DN)^{2}}\biggr]\xi + \biggl[ - d^{2\alpha } f^{\alpha } D + d^{2\alpha } f^{\alpha } N + d^{3\alpha } \\ &\quad{} + \frac{a^{\alpha } b^{\alpha } \theta ^{\alpha } \lambda ^{\alpha } d^{\alpha } N}{(1 + \theta ^{\alpha } A)^{2}(b^{\alpha } + DN)^{2}}\biggr] = 0. \end{aligned} $$

Define

$$\begin{aligned}& a_{3} = 1, \\& a_{2} = f^{\alpha } N - f^{\alpha } D + 3d^{\alpha }, \\& a_{1} = 2d^{\alpha } f^{\alpha } N - 2d^{\alpha } f^{\alpha } D + 3d^{2\alpha } + \frac{a^{\alpha } b^{\alpha } \theta ^{\alpha } \lambda ^{\alpha } N}{(1 + \theta ^{\alpha } A)^{2}(b^{\alpha } + DN)^{2}}, \\& a_{0} = - d^{2\alpha } f^{\alpha } D + d^{2\alpha } f^{\alpha } N + d^{3\alpha } + \frac{a^{\alpha } b^{\alpha } \theta ^{\alpha } \lambda ^{\alpha } d^{\alpha } N}{(1 + \theta ^{\alpha } A)^{2}(b^{\alpha } + DN)^{2}}. \end{aligned}$$

According to the Routh–Hurwitz criterion [21], the stable conditions are $a_{3} > 0$, $a_{2} > 0$, $a_{1} > 0$, $a_{0} > 0$, and $a_{2}a_{1} - a_{3}a_{0} > 0$.

#### Proof

$a_{3} = 1 > 0$ is obviously true.

$$ \begin{aligned} a_{2} &= f^{\alpha } N - f^{\alpha } D + 3d^{\alpha } \\ &= f^{\alpha } N - \frac{f^{\alpha } d^{\alpha } N - f^{\alpha } \lambda _{N}}{d^{\alpha } N} + 3d^{\alpha } = \frac{f^{\alpha } d^{\alpha } N^{2} + (3d^{2\alpha } - f^{\alpha } d^{\alpha } )N + f^{\alpha } \lambda _{N}}{d^{\alpha } N}. \end{aligned} $$

If $(3d^{2\alpha } - f^{\alpha } d^{\alpha } )^{2} - 4f^{2\alpha } d^{\alpha } \lambda _{N}^{\alpha } < 0$, namely $\frac{9d^{3\alpha } + f^{2\alpha } d^{\alpha }}{4f^{2\alpha } \lambda _{N}^{\alpha } + 6f^{\alpha } d^{2\alpha }} < 1$, we have $a_{2} > 0$. When $\frac{d^{3\alpha } + f^{2\alpha } d^{\alpha }}{4f^{2\alpha } \lambda _{N}^{\alpha } + 2f^{\alpha } d^{2\alpha }} < 1$, $a_{3} > 0$, $a_{2} > 0$, $a_{1} > 0$, $a_{0} > 0$.

$$\begin{aligned}& a_{2}a_{1} - a_{3}a_{0} \\& \quad = \bigl(f^{\alpha } N - f^{\alpha } D + 3d^{\alpha } \bigr) \times \biggl[2d^{\alpha } f^{\alpha } (N - D) + 3d^{2\alpha } + \frac{a^{\alpha } b^{\alpha } \theta ^{\alpha } \lambda ^{\alpha } N}{(1 + \theta ^{\alpha } A)^{2}(b^{\alpha } + DN)^{2}}\biggr] \\& \quad\quad{} + d^{2\alpha } f^{\alpha } D - d^{2\alpha } f^{\alpha } N - d^{3\alpha } - \frac{a^{\alpha } b^{\alpha } \theta ^{\alpha } \lambda ^{\alpha } d^{\alpha } N}{(1 + \theta ^{\alpha } A)^{2}(b^{\alpha } + DN)^{2}} > 0 \\& \quad = 2d^{\alpha } f^{2\alpha } N^{2} - 2d^{\alpha } f^{2\alpha } DN + 3d^{2\alpha } fN + \frac{a^{\alpha } b^{\alpha } \theta ^{\alpha } \lambda ^{\alpha } f^{\alpha } N^{2}}{(1 + \theta ^{\alpha } A)^{2}(b^{\alpha } + DN)^{2}} - 2d^{\alpha } f^{2\alpha } DN \\& \quad\quad{} + 2d^{\alpha } f^{2\alpha } D^{2} - 3d^{2\alpha } f^{\alpha } D - \frac{a^{\alpha } b^{\alpha } \theta ^{\alpha } \lambda ^{\alpha } f^{\alpha } DN}{(1 + \theta ^{\alpha } A)^{2}(b^{\alpha } + DN)^{2}} + 6d^{2\alpha } f^{\alpha } N - 6d^{2\alpha } f^{\alpha } D + 9d^{3\alpha } \\& \quad\quad{} + \frac{3a^{\alpha } b^{\alpha } \theta ^{\alpha } \lambda ^{\alpha } d^{\alpha } N}{(1 + \theta ^{\alpha } A)^{2}(b^{\alpha } + DN)^{2}} + d^{2\alpha } f^{\alpha } D - d^{2\alpha } f^{\alpha } N - d^{3\alpha } - \frac{a^{\alpha } b^{\alpha } \theta ^{\alpha } \lambda ^{\alpha } d^{\alpha } N}{(1 + \theta ^{\alpha } A)^{2}(b^{\alpha } + DN)^{2}} > 0 \\& \quad = 2d^{\alpha } f^{2\alpha } N^{2} + 8d^{2\alpha } f^{\alpha } N - 4d^{\alpha } f^{2\alpha } DN + 2d^{\alpha } f^{2\alpha } D^{2} - 8d^{\alpha } f^{2\alpha } D + 8d^{3\alpha } \\& \quad\quad{} + \frac{a^{\alpha } b^{\alpha } \theta ^{\alpha } \lambda ^{\alpha } (f^{\alpha } N^{2} - f^{\alpha } DN + 2d^{\alpha } N)}{(1 + \theta ^{\alpha } A)^{2}(b^{\alpha } + DN)^{2}} > 0 \\& \quad = 2d^{\alpha } \bigl[f^{2\alpha } (N - D)^{2} + 4d^{\alpha } f^{\alpha } (N - D) + 4d^{2\alpha } \bigr] + \frac{a^{\alpha } b^{\alpha } \theta ^{\alpha } \lambda ^{\alpha } (f^{\alpha } N^{2} + d^{\alpha } N + \lambda _{N}^{\alpha } )}{(1 + \theta ^{\alpha } A)^{2}(b^{\alpha } + DN)^{2}} \\& \quad = 2d^{\alpha } \bigl[f^{\alpha } (N - D) + 2d^{\alpha } \bigr]^{2} + \frac{a^{\alpha } b^{\alpha } \theta ^{\alpha } \lambda ^{\alpha } (f^{\alpha } N^{2} + d^{\alpha } N + \lambda _{N}^{\alpha } )}{(1 + \theta ^{\alpha } A)^{2}(b^{\alpha } + DN)^{2}} > 0. \end{aligned}$$

Therefore, $a_{2}a_{1} - a_{3}a_{0} > 0$. Using the Routh–Hurwitz criterion [21], when $\frac{d^{3\alpha } + f^{2\alpha } d^{\alpha }}{4f^{2\alpha } \lambda _{N}^{\alpha } + 2f^{\alpha } d^{2\alpha }} < 1$, $\vert \arg (S_{1,2,3}) \vert > \frac{\alpha \pi }{2}$, equilibrium $E_{1} = (D_{1},N_{1},A_{1},D_{2},N_{2},A_{2})$ is locally asymptotically stable.

**Figure 4:**
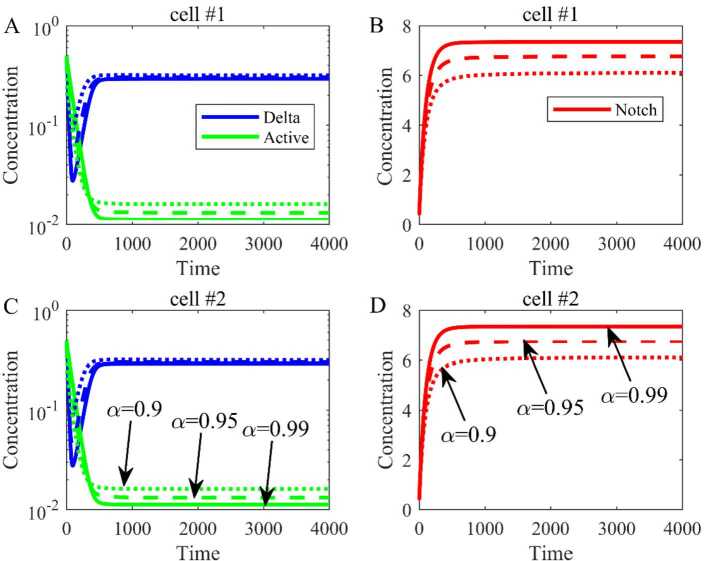
The dynamical trend of Delta, Notch, and active Notch when $\lambda \ne 0$. (**A**) The dynamical trend of Delta and active Notch in cell 1. (**B**) The dynamical trend of Notch in cell 1. (**C**) The dynamical trend of Delta and active Notch in cell 2. (**D**) The dynamical trend of Notch in cell 2

All the parameters and initial values are the same except $\lambda = 1000$. The equilibrium is $E_{1} = (0.2349, 6.1065, 0.0405, 0.2349, 6.1065, 0.0405)$ and the simulation results are shown in Fig. 4. Similarly, when the order (*α*) of fractional differential equations varies from 0.9 to 0.99, the trends are the same, and the equilibrium is bigger with the increase of order. This suggests that the order of fractional differential equation can affect the equilibrium.

□

## Sensitivity analysis

Sensitivity analysis is a method to identify critical inputs (parameters) of a model and quantify how input uncertainty impacts model outcome [25]. We conduct sensitivity analysis to investigate the significance of parameters by the Morris method. The basic idea is to assess the change in the response output caused by a small variation of parameter.

### Sensitivity values of eight parameters in the two cell model

Assume that the base effect of a model can be represented as the following equation:

15 $$ d_{i}(j) = \frac{f ( x_{1},x_{2},\ldots,x_{i - 1},x_{i} + \Delta ,x_{i + 1},\ldots,x_{n} ) - f ( x_{1},\ldots,x_{n} )}{\Delta }, $$

where $d_{i}(j)$ is the base effect of the *i*th parameter in *j* group ($j = 1,2,3,\ldots,R$). *R* is the number of repeated sampling. *n* is the number of parameters. $x_{i}$ is the *i*th parameter, and Δ is the small variation of parameter. $f(\bullet )$ is the response output. The sensitivity can be calculated by the following equation:

16 $$ S_{i} = \frac{1}{R}\sum_{j = 1}^{R} \bigl\vert d_{i}(j) \bigr\vert . $$

The sensitivity values of eight parameters are shown in Table 2.

**Table 2:** The sensitivity values of eight parameters

Parameters	*λ*	*f*	*d*	$\lambda _{N}$	*a*	*b*	*α*	*θ*
$S_{i}$	5.27e−05	2.233	18.933	1.941	1.424	2.263	2.864	2.32e−07

### Sensitivity test in the two cell model

**Figure 5:**
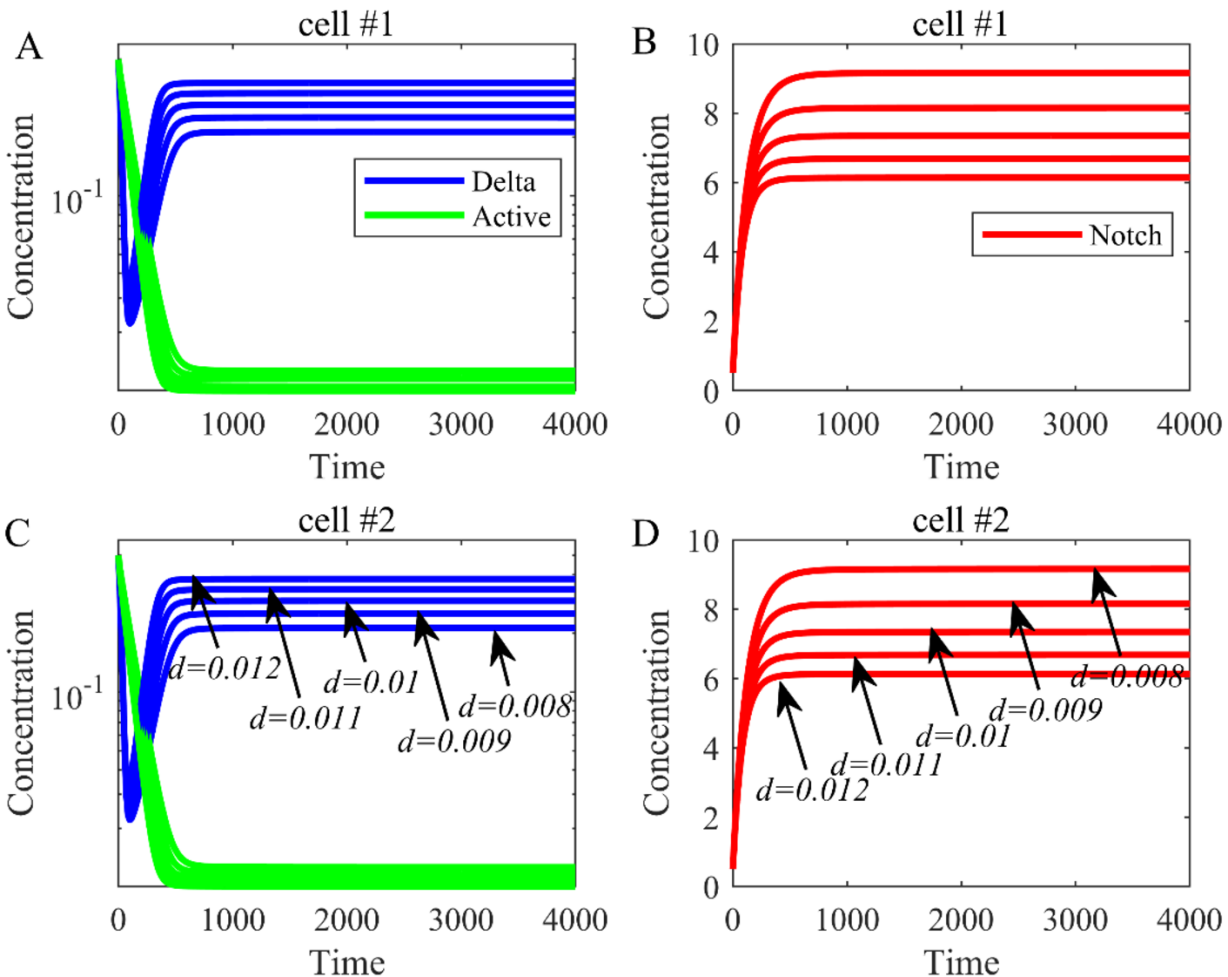
The sensitivity test of *d* from 0.008 to 0.012. (**A**) The dynamical trend of Delta and active Notch in cell 1. (**B**) The dynamical trend of Notch in cell 1. (**C**) The dynamical trend of Delta and active Notch in cell 2. (**D**) The dynamical trend of Notch in cell 2

In this subsection, we test the sensitivity of parameters by numerical simulation. Firstly, we verify parameters *d* and *λ* in the two cell model. Based on Table 1, the parameter *d* is 0.01, and we change *d* from 0.008 to 0.012 with a step 0.001. The results as shown in Fig. 5 illustrate that parameter *d* with small perturbations can have a large effect on the output of Delta ligand (blue line) and Notch receptor (red line) in the two cell model.

**Figure 6:**
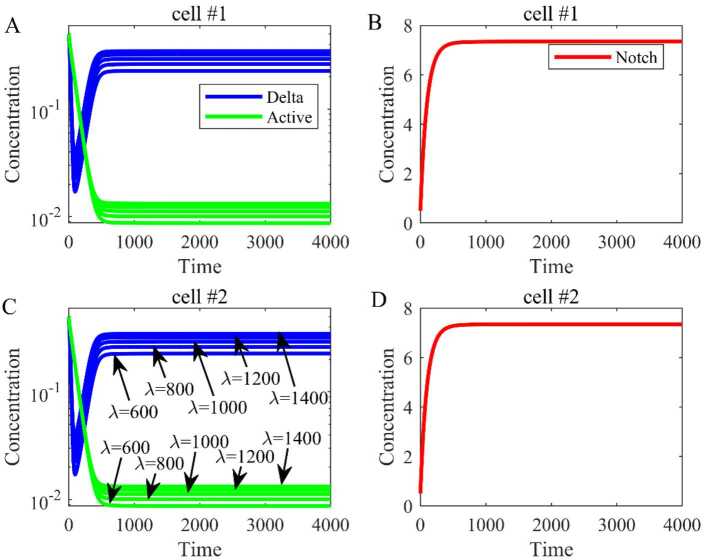
The sensitivity test of *λ* from 600 to 1400. (**A**) The dynamical trend of Delta and active Notch in cell 1. (**B**) The dynamical trend of Notch in cell 1. (**C**) The dynamical trend of Delta and active Notch in cell 2. (**D**) The dynamical trend of Notch in cell 2

Similarly, the parameter *λ* is 1000 at the beginning, and we change it from 600 to 1400 with a step 200. The results as shown in Fig. 6 suggest that there is no obvious change in Notch receptor (red line) and only a little change in Delta ligand (blue line).

According to the numerical simulations above, the sensitive parameter can significantly affect the expression of Delta ligands and Notch receptors, while the insensitive parameter cannot.

### Sensitivity test in 60 cells

Based on the sensitivity analysis in two cells, a 60 cell model with 180 dimensional fractional-order differential equations has been verified using numerical simulation.

#### Phenotype changes due to parameter *d* changes

**Figure 7:**
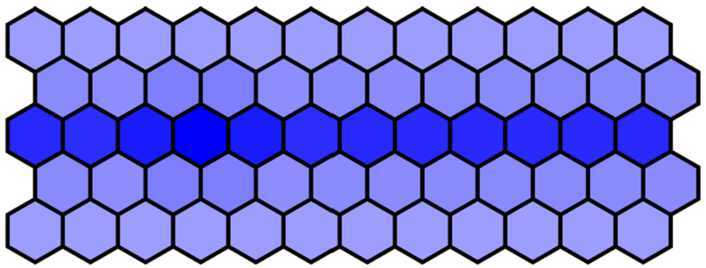
The wild-type phenotype dyed in deep color in the middle row and in light color in others when $d = 0.018$

Firstly, 60 cells were arranged into 5 rows × 12 columns, and the parameter *λ* was defined $\lambda = 1000$ in the first three rows, $\lambda = 0$ in the fourth and fifth rows. Other parameters were chosen as in Table 1 except $d = 0.018$. Blue intensity denotes the expression of Notch levels. Then, we get the wild-type phenotype dyed in deep color in the middle row and in light color in others. The wild-type phenotype obtained from the numerical simulation as shown in Fig. 7 is consistent with the experimental findings (Fig. 1(a)).

**Figure 8:**
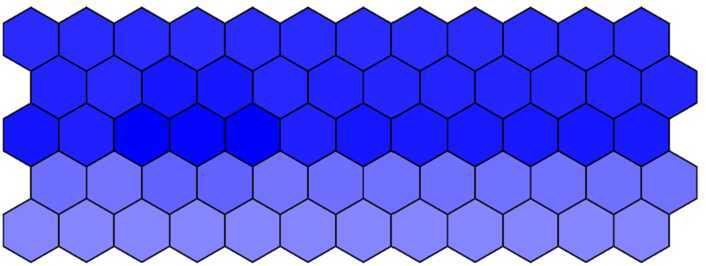
The mutant phenotype dyed in deep color in the first three rows and in light color in the fourth and fifth rows when $d = 0.012$

**Figure 9:**
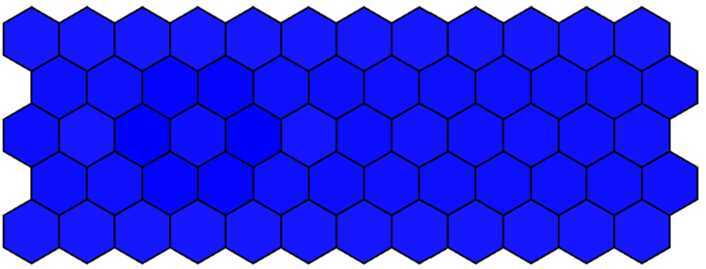
The complete mutant phenotype dyed in deep color in all five rows when $d = 0.001$

Then, we decrease *d* from 0.018 to 0.001 with a step 0.001 and run simulation to obtain the simulation results. When $d = 0.012$ and other parameters remain unchanged, we get the mutant phenotype the first three rows of which are dyed in deep color and the fourth and fifth rows in light color with over-expressed Notch in the first three rows. The mutant phenotype is shown in Fig. 8 and is consistent with experimental findings (Fig. 1(b)). When $d = 0.001$, the Notch in five rows is all over-expressed, and then five rows are all dyed in deep color. The complete mutant phenotype is shown in Fig. 9 and is consistent with the experimental findings (Fig. 1(c)).

So far, we have obtained the phenotypes of all the current experimental results through numerical simulation by changing sensitive parameter *d*. This also indirectly shows that the model established in this paper is effective.

#### Phenotype changes due to parameter *λ* changes

In this subsection, we research how phenotype changes due to parameter *λ* changes. Firstly, fix $d = 0.018$ and gradually increase *λ* from 1000 to 2000 with a step 200 and then run simulation to obtain the simulation results.

**Figure 10:**
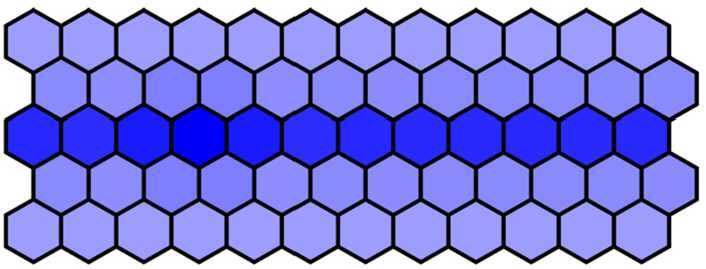
The wild-type phenotype dyed in deep color in the middle row and in light color in others when $\lambda = 1000$

**Figure 11:**
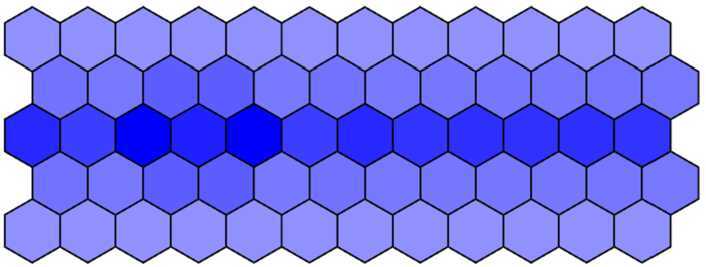
The wild-type phenotype dyed in deep color in the middle row and in light color in others when $\lambda = 1500$

**Figure 12:**
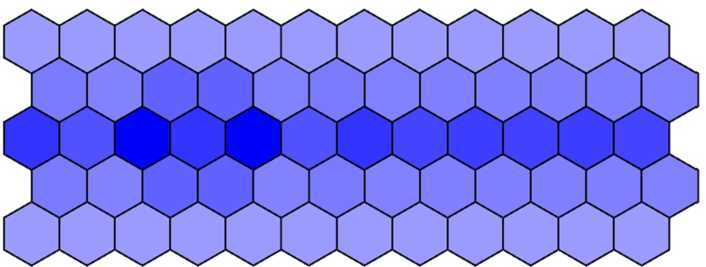
The wild-type phenotype dyed in deep color in the middle row and in light color in others when $\lambda = 2000$

It seems intuitively clear that all phenotypes (Figs. 10–12) are similar because they are all dyed in deep color in the middle row and in light color in others when we increase *λ* from 1000 to 2000. This also indirectly indicates that the effect of the parameter *λ* on the phenotype is not significant.

In conclusion, the verification of sensitivity analysis above shows that sensitive parameter *d* can obviously influence the phenotype, while relatively insensitive parameter *λ* cannot. This suggests the sensitivity analysis in our model is reliable, and we can minorly adjust the sensitive parameters to obtain ideal phenotypes.

## Comparison between the fractional-order model and the integer-order model

In this section, the comparison is done between the fractional-order model and the integer-order model in two cells and 60 cells models.

### The comparison in two cells

**Figure 13:**
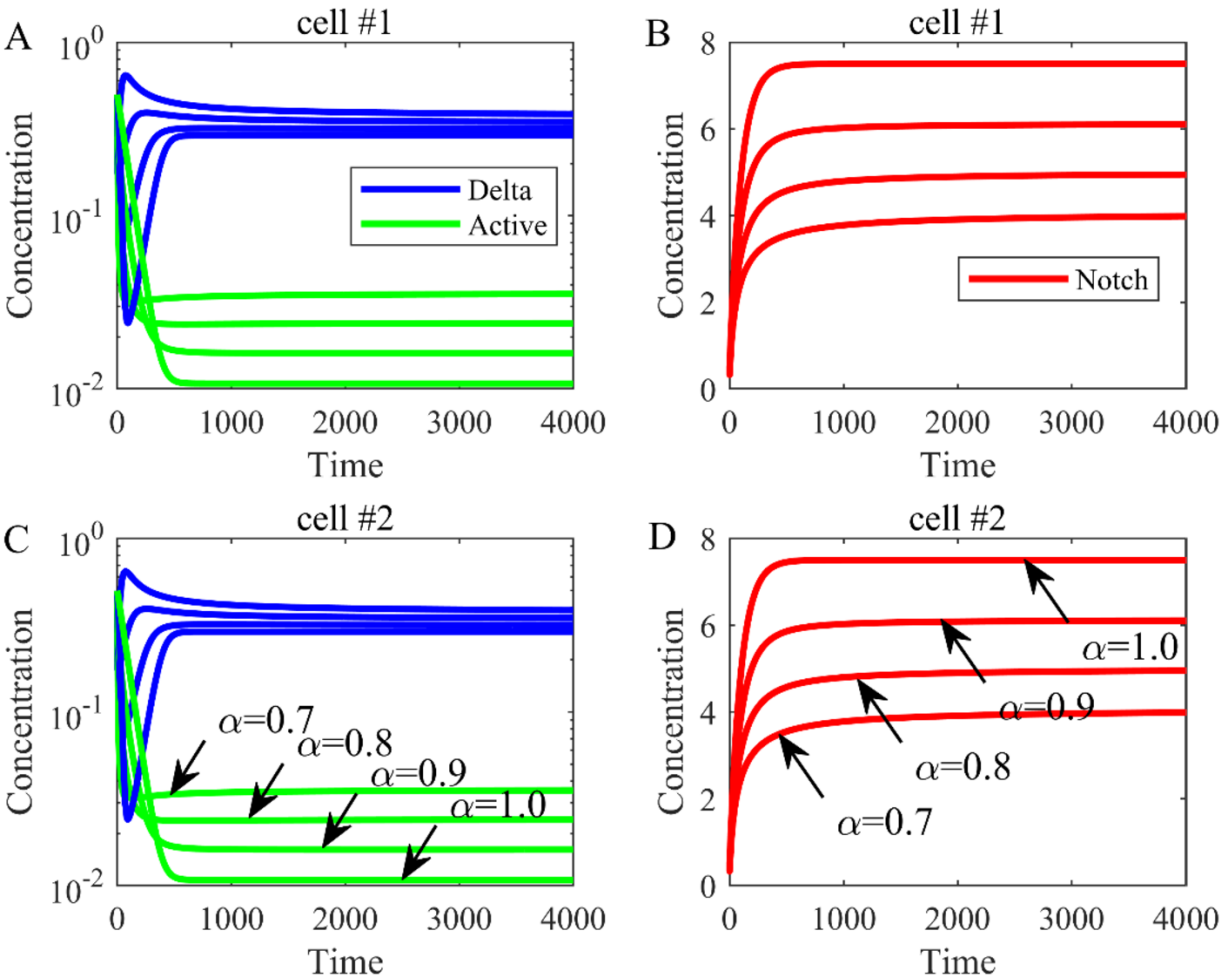
The dynamic between the fractional-order model and the integer-order model in two cells. (**A**) The dynamical trend of Delta and active Notch in cell 1. (**B**) The dynamical trend of Notch in cell 1. (**C**) The dynamical trend of Delta and active Notch in cell 2. (**D**) The dynamical trend of Notch in cell 2

Firstly, the dynamic between the fractional-order model and the integer-order model in two cells is compared, where the order is $\alpha = 0.9,0.8,0.7$ in the fractional-order model and $\alpha = 1$ in the integer-order model (Fig. 13). The simulation results show that under the same parameter value, although both the fractional-order model and the integer-order model reach the equilibrium, the equilibrium point is different. For instance, when $\alpha = 0.9$ the equilibrium of the fractional-order model is $(0.2349,6.1065,0.0405,0.2349,6.1065,0.0405)$ and the equilibrium of the integer-order model is $(0.2073,7.5000,0.0302,0.2073,7.5000, 0.0302)$ when $\alpha = 1$.

### The comparison in 60 cells

**Figure 14:**
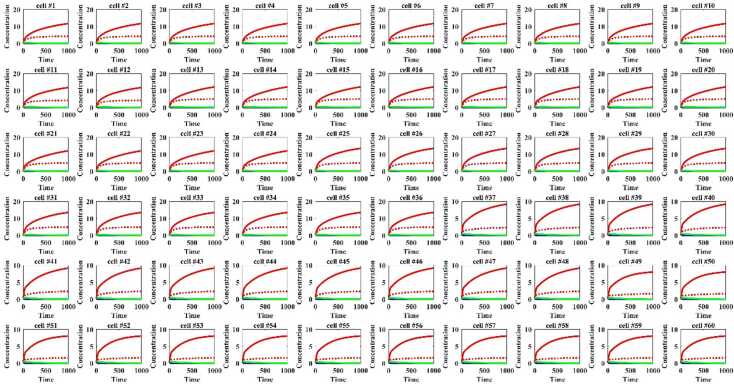
The dynamic between the fractional-order model (the dotted lines) and the integer-order model (the solid line) in 60 cells

In this part, the dynamic between the fractional-order model and the integer-order model in 60 cells is studied to explore how orders affect the phenotype. Similar to the situation of two cells, the dynamic trends of 60 cells are studied firstly. The results show that compared to the integer-order model (the solid line), the equilibrium of the integer-order model (the dotted lines) is obviously smaller (Fig. 14).

**Figure 15:**
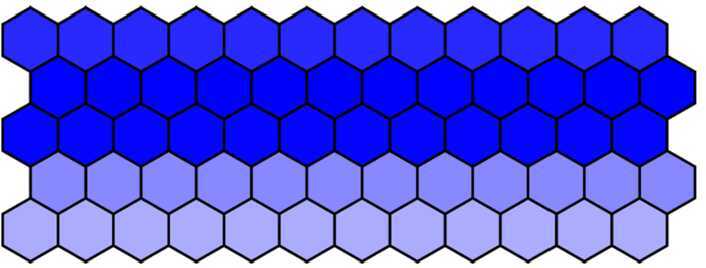
The mutant phenotype dyed in deep color in the first three rows and in light color in the fourth and fifth rows when $\alpha = 0.7$ and $d = 0.004$

**Figure 16:**
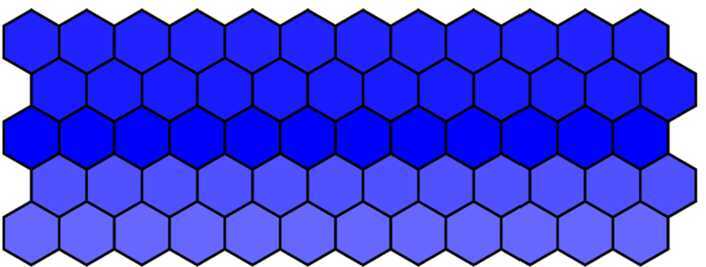
The mutant phenotype dyed in deep color in the first three rows and in medium color in the fourth and fifth rows when $\alpha = 1$ and $d = 0.004$

Next, the phenotypes have been analyzed between the fractional-order model and the integer-order model in 60 cells. In this part, we only studied the effect of parameter *d* changes on the phenotype, and the method of other parameters is similar. When $\alpha = 0.7$ and $d = 0.004$, the first three rows were dyed in deep color and the fourth and fifth rows were dyed in light color (Fig. 15). If $\alpha = 1$ and $d = 0.004$, the first three rows were dyed in deep color and the fourth and fifth rows were dyed in medium color (Fig. 16). Therefore, it is necessary to study the orders because fractional order can result in different phenotypes.

## Conclusion

In this paper, an improved mathematical model based on fractional-order differential equations for the Delta-Notch dependent boundary formation in the *Drosophila* large intestine was proposed for the first time. Because Notch signaling pathway is highly conserved in evolution and has significant hereditary properties, fractional differential equation which can better describe the memory characteristics and historical dependence of biological systems was used. We then calculated two equilibriums and studied the local asymptotic stability and also numerically illustrated the stability. Based on numerical simulation in the two cells model, we found that the order of the fractional-order differential equation can significantly affect the equilibrium point.

Moreover, parameter sensitivity analysis showed that different parameters have different sensitivities. The most and least sensitive parameters in the two cells model and the 60 cells model were verified by numerical simulations. The results demonstrated that a small change of sensitive parameter can significantly affect phenotype, while insensitive parameters cannot. Based on our established model, sensitivity analysis can help us to explore key parameters which can obviously affect phenotype, and we can get the ideal phenotype by adjusting these sensitive parameters.

Finally, the comparison was done between the fractional-order model and the integer-order model in two cells and 60 cells models. The results showed that the equilibriums and phenotypes of the fractional-order model are actually different from those of the integer-order model. For example, the expression of Notch is higher than that in the fractional-order model.

In the following, we will do some experiments and estimate an appropriate fractional order by the actual experimental data. What is more, we will compare and evaluate the fitting effects between the fractional-order model and the integer-order model.
